# Recycling of Waste Corundum Abrasive Powder in MK-Based Geopolymers

**DOI:** 10.3390/polym14112173

**Published:** 2022-05-27

**Authors:** Giovanni Dal Poggetto, Antonio D’Angelo, Michelina Catauro, Luisa Barbieri, Cristina Leonelli

**Affiliations:** 1 Department of Engineering “Enzo Ferrari”, University of Modena and Reggio Emilia, 41125 Modena, Italy; luisa.barbieri@unimore.it (L.B.); cristina.leonelli@unimore.it (C.L.); 2 Department of Engineering, University of Campania “Luigi Vanvitelli”, 81031 Aversa, Italy; antonio.dangelo@unicampania.it (A.D.); and michelina.catauro@unicampania.it (M.C.); 3 Department of Environmental, Biological and Pharmaceutical Sciences and Technologies, University of Campania “Luigi Vanvitelli”, 81100 Caserta, Italy

**Keywords:** geopolymer, corundum abrasive powder, recycling, metakaolin, microstructure, mechanical properties

## Abstract

Recycling corundum abrasive powder in metakaolin-based geopolymer formulations is proposed to reduce the amount of waste to be treated or disposed of in landfills, allowing to decrease ecological damage as well as to reduce transport costs for removal. The addition of waste corundum, as an important source of Al_2_O_3_, has proved to increase the slight ionic conductivity of the leachate solution obtained after immersion in water of samples at 28 d of curing at room temperature. With the same curing conditions, the geopolymerization process has not been disturbed as evidenced by the FT-IR peak shift and XRD patterns. It was recorded a decrease in resistance to compression of the consolidated geopolymers of about 5% with 10 wt% addition and of about 77% with the addition of 20 wt% of waste corundum. In any case, the waste abrasive powder does not release heavy metals when added to a geopolymeric formulation based on MK, NaOH, and Na-silicate, and does not show relevant antibacterial properties, indicating the formation of a stable and safe final product with a ceramic-like appearance.

## 1. Introduction

Nearly 2.5 trillion tonnes of materials were extracted and used globally from 1970–2017; of this, 44% were in excess of the sustainable corridor, as defined in [1]. The remaining parts are transformed into materials or energy. Of the total weight of released materials, about 60% was taken to landfill sites or sites for inert waste while the rest was recycled or reused [2]. Capable of saving both energy and minerals with respect to conventional binders, alkaline-activated materials are increasing their scientific interest and industrial applications. Among these, the class identified by Davidovits as geopolymers is distinguished by the near absence of CaO [3]. Actually, the term “geopolymer” was introduced by Davidovits in the 1970s, to identify novel inorganic binders with a content of SiO_2_+Al_2_O_3_ higher than 80 wt%, mostly X-ray amorphous, and commonly synthesized at room or slightly higher temperatures by reaction of a solid aluminosilicate powder with a concentrated alkali metal silicate or hydroxide solution [3,4]. The role of CaO is nevertheless relevant as reported by several authors [5] as other classifications have been proposed [6,7,8], but these issues depart from the aim of this present study.

Among the aluminosilicate sources, metakaolin (Al_2_O_3_·2SiO_2_) certainly plays an important role as a reference material being a source of pure alumina and silica with very low levels of impurities [4,9]. In addition to the activating solution, often based on NaOH and sodium silicate, more or less reactive fine aggregates are added, with the aim of evaluating their effects on the chemical, physical, morphological, and mechanical properties of the final solid [10].

In literature there are numerous studies reporting the addition of:

(1) SiO_2_ sand, even beach sand, to refine their rheological and mechanical resistance to compression combined with a decrease in drying shrinkage [11,12];
(2) rice hush ash, bagasse ash, and other ashes from biomasses rich in silica to decrease curing time and increase mechanical properties [13];
(3) alpha or gamma-alumina to increase the overall refractoriness of the binder [14,15];
(4) a combination of Al_2_O_3_ plus SiO_2_ sources in form of simple oxide mixture to increase density and thermal resistance [16];
(5) ultra-fine slag to increase in strength characteristics and improve durability [17];
(6) waste glass from containers and WEEE (Waste Electrical and Electronic Equipment) recycling to decrease shrinkage upon drying and toughness [18]; and
(7) ceramic filler to improve dimensional stability and mechanical resistance after thermal exposure [19].

In this study, corundum powder recovered from abrasimeter was used as the main alumina resource. This powder, indicated as recycled corundum (RC), even though crystalline, with its contribution of alumina to the metakaolin formulation, can act as a semi-reactive filler, as already highlighted in the literature [20]. It is well known that in a strong alkaline solution, aluminosilicate sources are rapidly dissolved to form free monomeric units of Si(OH)_4_ and Al(OH)_4_^−1^ tetrahedra. With the development of the reticulation reaction between these two species, water molecules deriving from the condensation of the –OH groups, are released from the geopolymeric network. Such a network is an alternation of SiO_4_ and AlO_4_ tetrahedral units linked to yield a 3D structure, or a linear one dependently upon Si/Al ratio [21]. RC could thus provide, even though partially, a number of Al^+3^ species in the alkaline solution to increment the Si/Al ratio. As proved in the paper of Riahi [20], this partial dissolution creates a strong interface between the MK-based binder and the Al_2_O_3_ particle capable of fracture-deviation reinforcement mechanisms. Notwithstanding this expected reactivity, we also assume that a large proportion of the corundum particles cannot dissolve, leaving an inert solid filler capable of resisting to compressive stress [14].

Our study, although focused on the substitution of metakaolin with RC in the formulation of NaOH and sodium silicate-based geopolymers, does not forget the nature of the waste. This waste, derived from abrasion tests of both metallic and ceramic materials, has the weakness of containing different components and possibly heavy metals, too. Therefore, as minor objectives, we set ourselves the control of the 3D network lattice of the geopolymer (integrity test, FT-IR), the verification of the reactivity of the crystalline phases (XRD) and, finally, the environmental impact (release of hazardous cations and antibacterial tests).

Apart from release of the three main components, Na^+^, Al^+3^, and Si^+4^, from the geopolymeric network [22] concerning the leaching of heavy metal cations, we expect a good encapsulating response from part of the geopolymeric matrix [23], thus obtaining a dense and safe binder suitable for the production of non-structural building materials, whereas the assessment of the antimicrobial activity could be useful to extend the possible application fields (glazes on ceramic tiles, pastes, etc.).

## 2. Materials and Methods

### 2.1. Materials

**Figure 1:**
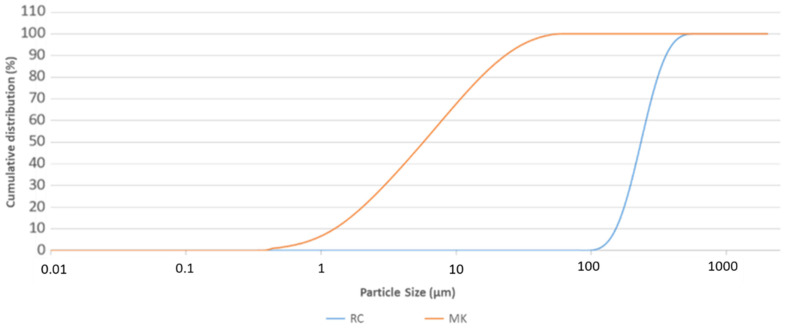
Comparison of particle size cumulative distribution curves of pure metakaolin (MK) and recycled corundum (RC).

The metakaolin (MK) used in this study was ARGICAL^TM^ M1000 (Imerys S.A.—43 Quai de Grenelle, 75015 Paris, France, with chemical composition reported by the producer: SiO_2_  =  55%; Al_2_O_3_  =  40%, Fe_2_O_3_  =  1.4%; TiO_2_  =  1.5%; Na_2_O  +  K_2_O  =  0.8%; CaO  +  MgO  =  0.3%; and LOI  =  1%). The high reactivity of this aluminosilicate precursor is due to its fine particle size (D50 = 8.2 µm; D90 = 33 µm particle size distribution (Figure 1), high surface area (B.E.T. = 17 m^2^/g [24] and appropriate Si/Al. Its use in literature as reference material for the geopolymer binders is very diffused [24].

The recycled corundum (RC) powder used in the lab scale abrasimeter (AP/87 Ceramic Instruments, Sassuolo, Italy) is a commercial product: White Corundum FEPA F80 (0.212–0.150 mm) (Ceramics Instruments, Sassuolo, Italy, Al_2_O_3_ 99%, 1% traces of MgO, Fe_2_O_3_, SiO_2_ and TiO_2_) used for abrasion test of different types of materials (ceramic, and glass) and was used as obtained. As it is well-known corundum is inert and does not have toxic and carcinogenic properties, it appears as a white and odorless powder, even after use on the abrasimeter. The declared F80 grain size, accordingly to FEPA-Standard 43-2:2017, RC’s particle size of 232 µm ((D50 = 232 µm; D90 = 300 µm) was confirmed by dynamic light scattering-DLS grain sizer Mastersizer 2000 (Malvern Instruments Ltd., Malvern, UK) and is reported in Figure 1 where the powder of the as-received metakaolin is presented, too. The chemical composition as determined by means of Energy Dispersive X-ray Fluorescence spectroscopy (EDXRF) is reported in Table 1. A Shimadzu Spectrometer EDX-720 (GmbH, Duisburg, Germany) equipped with 50 W Rh target X-ray tube, a high-energy resolution Si (Li) detector, and five primary X-ray filters, was used. Being a waste, the RC was checked for the eventual leachability of heavy metals according to the EN 12457-2:2004; details are reported in Section 2.3.2. Leaching Test.

Additionally, RC was checked for its reactivity in alkaline environment after immersion in different NaOH solutions. To choose the most reactive concentration of NaOH for RC to be used for geopolymer formulations, we carried out 4 tests on the recycled corundum powders using increasing NaOH concentrations: 6, 8, 10, and 12 M. The NaOH solutions were prepared by dissolving laboratory-grade granules (96 wt%, Sigma-Aldrich, Italy) into distilled water to have 6, 8, 10, and 12 M concentrations.

The sodium silicate solution (SiO_2_/Na_2_O = 3.00 molar ratio; SiO_2_ = 26.50 wt%, Na_2_O = 8.70 wt%, and pH = 11.7) with bulk density of 1.34 at 20 °C was used in the formulation of the geopolymers in combination with NaOH. The solution of sodium silicate was provided by Ingessil, Verona, Italy.

### 2.2. Preparation of Geopolymer Specimens

From the results of the alkali activation of RC at different NaOH concentrations (see comments in Section 3.4 and XRD results shown in Figure 5) we have chosen to use the NaOH 8M.

The reference geopolymer, hereafter indicated as GP0, was obtained by adding 30 mL of NaOH, 8M plus 30 mL of sodium silicate to the amount of 100 g of MK, dry powder, under mechanical stirring. In the GP0 formulation, the MK powder was substituted by 10 and 20 wt% of as-received recycled corundum powder to make the geopolymer composites labelled GP-10RC and GP-20RC, respectively.

All geopolymers were prepared through Planetary Mixer (Aucma 1400W, China). The fresh paste was poured into silicon cube molds (25 mm × 25 mm × 25 mm). After removing all the bubbles with the vibrating table, the molds were attentively closed and geopolymers cured at room temperature at 100% relative humidity. The silicon molds were opened after 28 d of curing time to proceed with the proper characterization. A minimum of 10 samples per each formulation were obtained.

### 2.3. Geopolymers Characterization

#### 2.3.1. Ionic Conductivity Measurements

In order to evaluate the overall chemical stability of the 3D aluminosilicate network reticulated within the geopolymer final products, the immersion in water was tested accordingly to ref. [25]. MilliQ water (1:10 = solid:water weight ratio) was added to the grounded geopolymer samples. After stirring the solution, a short time was waited to sediment the solids before analyses. Ionic Conductivity measurements were performed on the eluates with Crison GLP31 (Hach Lange Spain, S.L.U, Barcelona, Spain), respectively, at t_1_ = 0 h, t_2_ = 5 min, t_3_ = 10 min, t_4_ = 30 min, t_5_ = 2 h, t_6_ = 4 h, t_7_ = 6 h, t_8_ = 24 h, and t_9_ = 48 h.

#### 2.3.2. Leaching Test

The ability to leach the ions of Al^+3^ and Si^+4^ deriving from an incomplete reticulation, as well as the release in water of heavy metals from recycled corundum powder as well as all the geopolymer formulations was checked according to the EN 12457-2:2004 (“Characterisation of Waste-Leaching-Compliance test for leaching of granular waste materials and sludges-Part 2: One stage batch test at a liquid to solid ratio of 10 L/kg for materials with particle size below 4 mm (without or with size reduction)”). After crushing and sieving to particle sizes minor than 4 mm, the geopolymeric samples were placed in bi-distilled water with 1:10 = solid:water weight ratio, and maintained for 24 h. After the recovery of the filtrated (d < 0.45 µm) leachates solutions, they were acidified with HNO_3_ (69%) solution to pH = 2. Then, according to EN ISO 11885:2009 (“Water Quality-Determination of selected elements by inductively coupled plasma optical emission spectrometry (ICP-OES)”), ionic heavy metal concentrations were determined by ICP-OES (Agilent, Santa Clara, CA, USA). All the ionic metal concentrations are expressed as ppm. The Limit Of Quantitation (LOQ) (the concentration at which imprecision (coefficient of variation) of the method is 5%) for Al, B, Ba, Fe, Mn, Sb, and Zn was 5 ppb, the Limit Of Detection (LOD) (the lowest concentration of the measurand that can be detected at a specified level of confidence) of Be, Cd, Co, Cr, Cu, Mo, Ni, Pb, Se, Sn, and V was 2 ppb, while the LOQ of Si and Ca was 500 ppb.

#### 2.3.3. Microstructural Characterization

With the aim to evaluate the presence of additional Al, deriving from the RC, into the geopolymer network, FT-IR analysis (Prestige21 Shimadzu spectrophotometer, Shimadzu Italia s.r.l. Via G.B. Cassinis, Milano equipped with a detector deuterated triglycine sulfate with potassium bromide windows) was performed on geopolymers and raw materials powders, either RC or MK. The analysis was performed in the range of 400–4000 cm^−1^ and with a resolution of 2 cm^−1^ (60 scans). For the analysis, KBr disks containing 2 mg of sample and 198 mg of KBr were used. FT-IR spectra were elaborated by IRsolution and Origin 9 softwares.

#### 2.3.4. Mineralogical Composition

Crystalline phases of the geopolymers and raw materials (MK and RC) were identified on powdered specimens via X-ray diffraction (XRD) (X’Pert PRO, PANAlytical, Malvern Panalyical Ltd., Malvern, UK) The diffractometer was operated at 40 kV and 40 mA using Cu-Kα radiation (Ni filtered). Diffraction patterns were collected by the X’Celerator detector from 5 to 70° 2θ with a step size of 0.02° 2θ and a counting time of 3 s. It should be noted that the as-received waste corundum powder was not finely ground to avoid contamination from milling media as it is an extremely hard abrasive (value of 9 on the Mohs hardness scale). Mineral phases were identified by comparing the experimental peaks with reference patterns (DIFFRAC plus EVA software, 2005 PDF2, Bruker, Billerica, MA, USA).

#### 2.3.5. Mechanical Properties

To evaluate the mechanical performance of the MK-based geopolymers with 0, 10% and 20 wt% of recycled corundum, compression tests were performed after 28 d of curing (Instron 5567 Universal Testing Machine, Norwood, MA, USA). For the tests, cubic samples 25 mm × 25 mm × 25 mm were used. The load (30 kN load limit) was applied and increased by displacement rate of 1 mm/min. The tests were executed in displacement control mode at a constant loading velocity and no preload. They were stopped after obtaining three valid tests for each different geopolymer composition. Compressive strength values are assumed to be the mean value of three tests attended with the 2% variance.

#### 2.3.6. Antibacterial Properties

To analyze the potential antibacterial activity, samples were incubated with four bacterial strains. *Escherichia coli* (American Type Culture Collection-ATCC^®^ 25922^™^, Manassas, VA, USA) and *Pseudomonas aeruginosa* (ATCC^®^ 27853^™^) as gram-negative bacteria and *Staphylococcus aureus* (ATCC^®^ 25923^™^) and *Enterococcus faecalis* (ATTC^®^ 29212^™^) as gram-positive bacteria.

After the samples were finely ground with a mortar and a pestle, they were sterilized under UV light for 1 h before the incubation with the bacterial strains. The bacterial suspensions of 10^9^ CFU/mL were obtained by diluting the pelletized strains in autoclaved bi-distilled saline water (0.9% of NaCl). After the bacterial dissolution; *E. coli* was plated on TBX Medium, (Tryptone Bile X-Gluc, Liofilchem, Italy), *S. aureus* on Baird-Parker agar (Liofilchem, Italy), *P. aeruginosa* on Pseudomonas CN Agar (Liofilchem, Italy) and, finally, *E. faecalis* on Slanetz Bartley agar base (Liofilchem, Italy). All media were sterilized up to 120 °C for 15 min. Before the incubation times; 100 mg of sample powders were placed in the middle of Petri dishes. *E. coli* and *S. aureus* plates were incubated, respectively, at 44 °C and 36 °C for 24 h, while *E. faecalis* and *P. aeruginosa* at 36 °C for 48 h. The diameter of inhibition halos (IDs) was calculated. Three replicates for each sample were performed to determine the average absolute deviation (AAD). All the entire procedure follows the one reported in [26].

## 3. Results

### 3.1. Ionic Conductivity

We have investigated the chemical stability and leaching behavior of RC-containing geopolymer matrices to assess the environmental characteristics and eventual risks.

Concerning the chemical stability of the aluminosilicate network it should be reminded that the higher reactivity of the alkaline solution with metakaolin, or with RC when present, the lower ionic and cationic release will be recorded.

**Figure 2:**
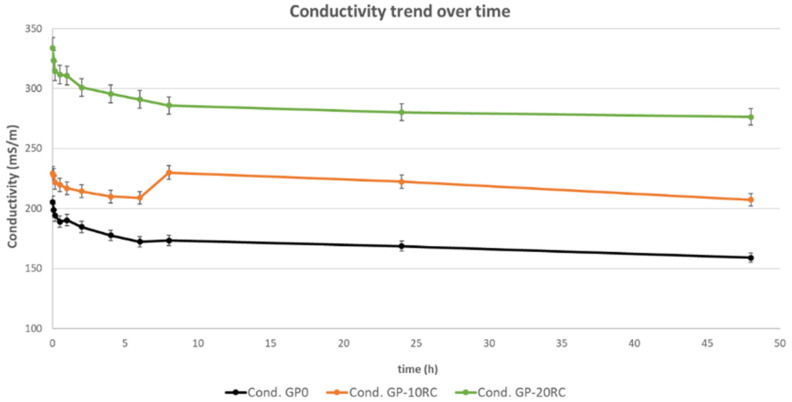
Ionic conductivity of the eluate (1:10 = solid:water weight ratio) vs. immersion time of the three geopolymers cured 28 d. (The reproducibility of the test was calculated to be within an error of 8%).

Regarding the pH analyses, the samples with 10% and 20% of RC have very similar behavior to the MK-based geopolymer (GP0). In fact, the pH is decreasing until it stabilizes after 48 h around the values of 10, being 9.86, 9.98, and 10.17 (error ± 2%) for GP0, GP10RC, and GP20RC, respectively. The situation is different for ionic conductivity analyses (Figure 2) that was stabilized after about 10 h, remaining almost constant or varying slightly for each analysed sample. The value differences found among the three geopolymers are in accordance with their pH values: the lower being recorded for GP0, indicating lowest ions release in solution. The reticulation of the 3D geopolymeric network has been confirmed also by the mechanical properties, as reported below in Figure 6 (Section 3.5).

### 3.2. Leaching Test

**Figure 3:**
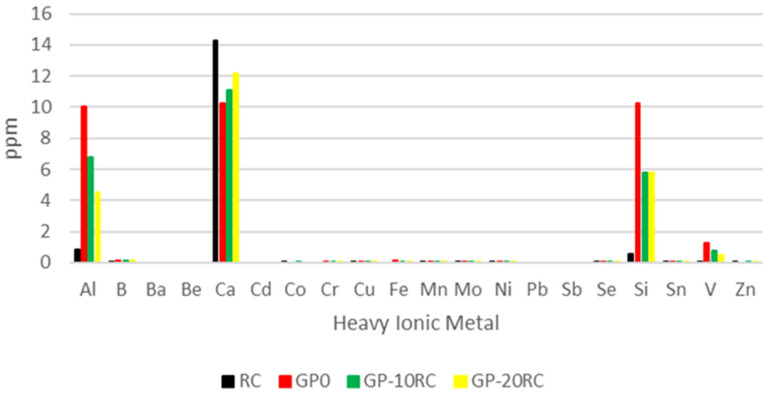
Results of the leaching test of the recycled corundum and the geopolymers samples. All the samples were diluted 1:10 and 1:20 to obtain the Al release. All the other heavy metals are detected without any dilution of the leachates.

The release of heavy ionic metals is reported in Figure 3. The histogram underlines that the mostly released heavy ionic metals are Al, Ca, Si, and V. All the other metals are present in traces. The aluminium ion is released at very low concentration by the RC, indeed the samples with 10 and 20% of RC release a lower amount of aluminium with respect to the GP0 sample. Similarly, GP0 releases the highest amount of silicon, being the Si-bearing impurities in RC not prone to leachate. This reflects also the decrease of silicon release passing from the GP-10RC and GP-20RC. Regarding the calcium release, it is higher for RC, and it decreases from GP-20RC to GP0. Finally, the vanadium release seems to depend on the amount of MK in the geopolymer samples, hence it is not brought into the geopolymeric matrix by the waste, but it is a contaminant of the natural mineral originating the metakaolin.

### 3.3. Microstructural Characterization

**Figure 4:**
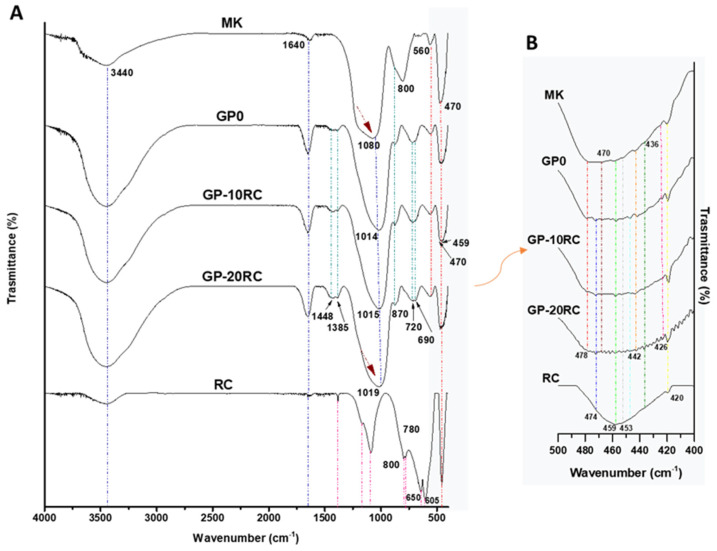
(**A**) FT-IR spectra of MK powder, GP0, GP-10RC, GP-20RC and RC powder. (**B**) Focus on 500–400 cm^−1^ IR range.

Figure 4 reports the FT-IR spectra of MK, RC powders, and the composite geopolymers GP-RC from 0 wt% to 20 wt%. All the spectra display the -OH stretching and bending at 3440 and 1640 cm^−1^. MK spectrum shows the Si-O-T (T = Si or Al) bands at 1080 cm^−1^. This peak is also recognized as DOSPM (Density of State Peak Maximum) and could be used to follow the geopolymerization [18,21,27]. Indeed, this band shifts to a lower wavenumber (1019–1014 cm^−1^) suggesting the increase of Si-O-Al bonds. Even though this shift seems to be slightly lower for the geopolymers with RC with respect to the reference GP0, the geopolymerization seems to not be affected by the recycled waste. In the spectrum of MK, the band at 800 cm^−1^ could be assigned to the presence of quartz, while the band at 560 cm^−1^ is related to Al-O vibration in six-fold coordination [28,29]. The band at 470 cm^−1^ in MK, GP (from 0 to 20 wt% of RC) is assigned to Si-OH bending mode (see also the comments on peak at 460 cm^−1^ from RC).

Regarding the RC spectrum, characterized by sharper bands as typical for crystalline compounds, it shows the main signal, in the shape of a sharp band, corresponding to the Al-O vibration at 1089 cm^−1^. According to [30,31], the presence of a peak at 1089 cm^−1^, 800, and 780 cm^−1^ are related to some disorders in the corundum network and could be attributed to the presence of additional type of coordination of the aluminium atoms (e.g., AlO_4_, and AlO_3_) [30]. The presence of the band at 459 cm^−1^ is assigned to Al-O bending. This band has a sharply intense shape in the recycled corundum spectrum, and, together with the band at 470 cm^−1^ became rounded but intense in the geopolymer samples with a higher amount of RC. Finally, GP0, GP-10 and 20RC show the bands at 1448–1385 cm^−1^ that are related to the formation of carbonates on their surfaces, maybe due to the interaction of sodium and calcium ions with the atmospheric CO_2_. These bands are higher for GP-10RC and GP-20RC, probably due to the presence of CaO in the RC as revealed by XRF analysis. This hypothesis is strengthened also by the presence of the band at 1384 cm^−1^ in the RC spectrum and the high release of calcium ions as observed in the leaching test. The enlargement in the IR range at 500–400 cm^−1^ (Figure 4B) confirmed the presence of vanadium, titanium, iron, and calcium as well as silicon and aluminium oxides. This band is more evident in MK and GP0, while is less intense in geopolymers with 10 and 20 wt% of RC. This follows also the data obtained by the leaching test, indeed, MK and GP0 showed the highest vanadium release than RC and GPs with RC. The medium broad band at 474 cm^−1^ (mainly visible for GP0, GP-10RC, GP-20RC, and as a shoulder in RC) is attributed to Al-O vibration, while the peaks at 453 and 442 cm^−1^ could be attributed to Fe-O vibrations [30]. Finally, Ti-O absorption broad bands are assigned to 436 and 426 cm^−1^ [30], whereas the sharp band at 420 cm^−1^ is assigned to Ca-O vibration [31].

### 3.4. Mineralogical Composition

**Figure 5:**
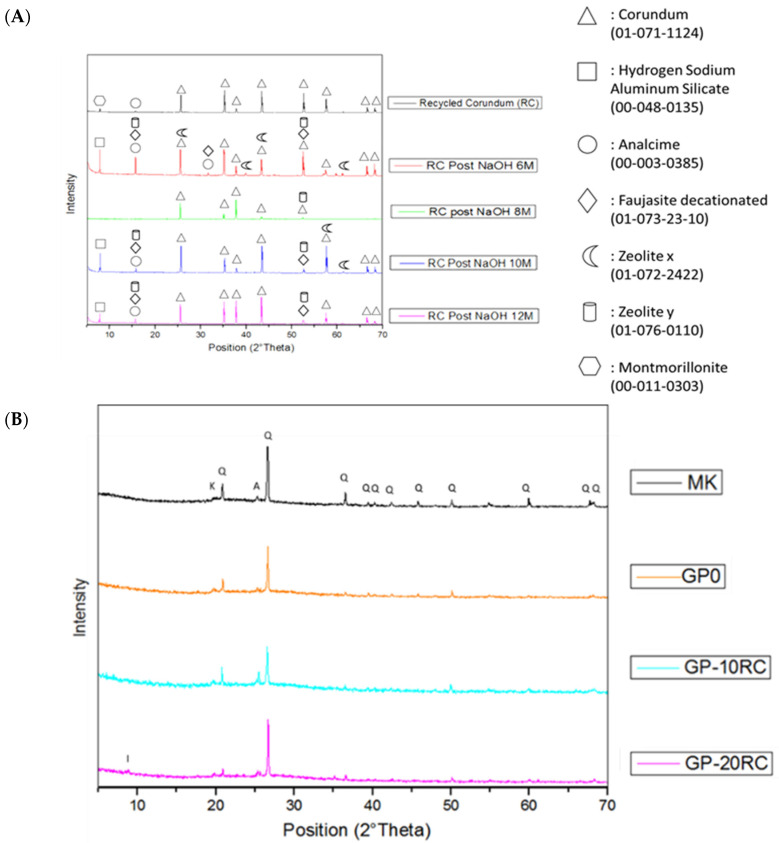

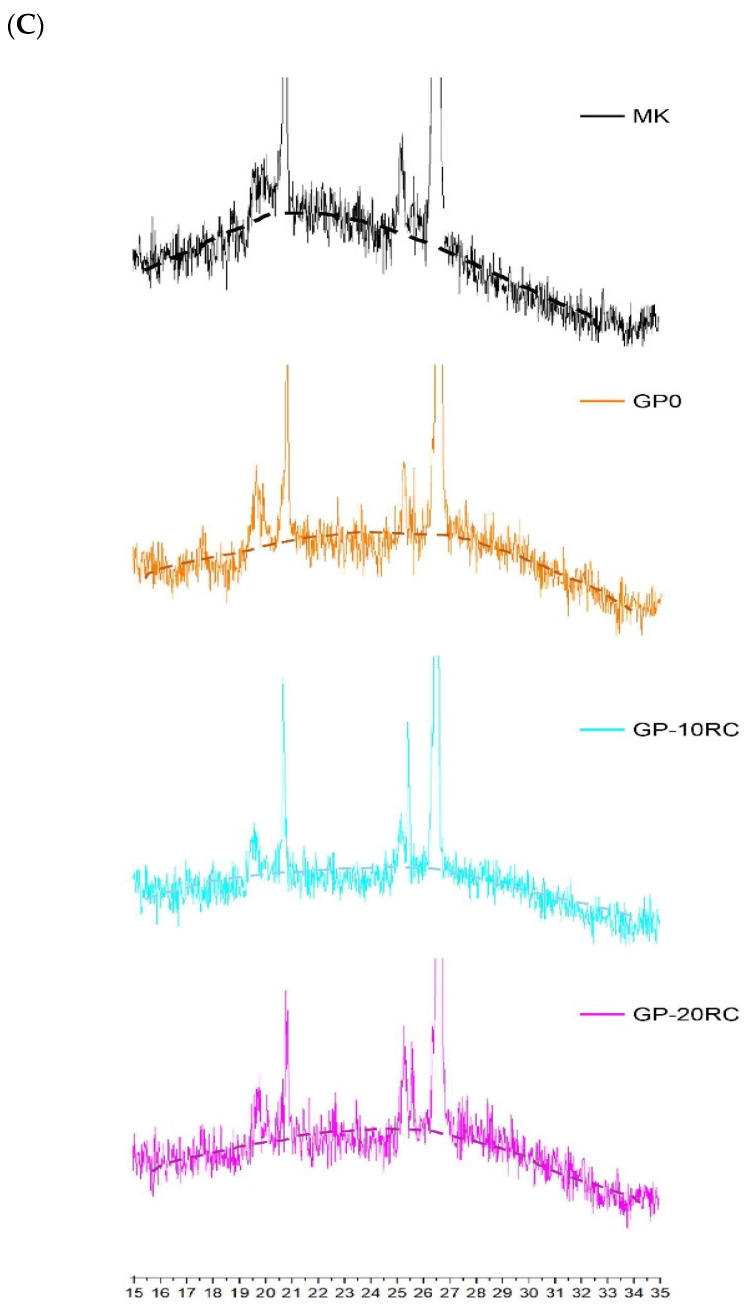
XRD patterns for: (**A**) RC raw materials and RC post basic attack of NaOH at different concentrations. (**B**) MK and GP0, GP-10RC and GP-20RC corundum based geopolymers. Crystalline phases identification label: Q = Quartz, K = Kaolinite, A = Anatase, I = Illite. (**C**) Enlargement of B spectra in the range 20–35° in 2 theta.

The mineralogical characterization of the recycled corundum abrasive powder before and after the NaOH attack (T = 85 °C) [32] was performed via XRD (Figure 5A).

Figure 5A compares the spectra of recycled corundum powder before and after basic NaOH attack at different concentrations. First of all, it must be noted that the spectrum of recycled corundum confirms what was seen at XRF, i.e., a high percentage of crystalline corundum, as typically obtained by the fusion of clean Al_2_O_3_ in electrical arc furnace, and small traces of other compounds most likely due to the use of this powder in the abrasimeter. It is observed that the powder after the attack of NaOH 6, 10, and 12 M did not dissolve very well, leading only to the formation of some phases.

On the other hand, the situation in the spectrum after the attack of NaOH 8 M is different since RC powder dissolves better, resulting in the disappearance of the peaks at 8° and 16° in 2θ (due to the impurities of the recycled corundum) and also at 57°, 68°, and 69° in 2θ typical of corundum.

In the diffraction pattern from metakaolin (Figure 5B) it is evident a diffuse reflection identified as the typical large band of the amorphous aluminosilicate structure plus shaper peaks identified as anatase, and alpha-quartz. The diffraction patterns from GP and GP-RC series were similar (Figure 5B,C), and all these three geopolymers had a diffuse reflections characteristic of amorphous aluminosilicate network at about 26–28° in 2θ [33]. By increasing the percentage of recycled corundum, a peak around 8° can be seen in the GP-20RC sample, which confirms the presence of recycled corundum that has not reacted perfectly, which is not noticeable in the GP-10RC spectrum due to the lower amount of RC.

### 3.5. Mechanical Properties

**Figure 6:**
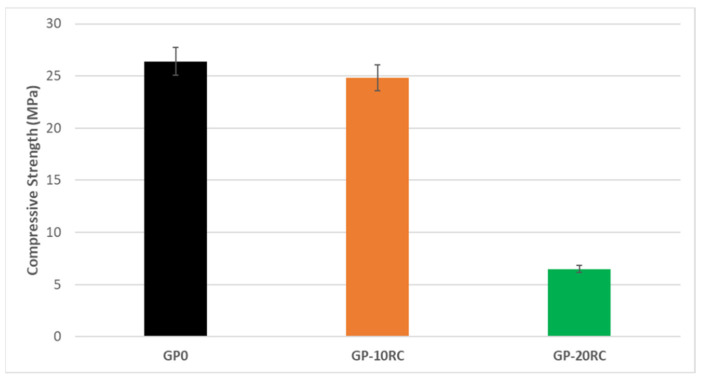
Compressive Strength for all the samples after 28 d.

The compressive strength tests were performed after 28 d of curing on all the geopolymer samples. From Figure 6 it is possible to notice that after an addition of 10% by weight of recycled corundum the value does not change particularly. The situation is different by adding 20% by weight of the raw material, as the compressive strength drops dramatically. A tentative explanation is provided in the Section 4.

### 3.6. Antibacterial Properties

**Figure 7:**
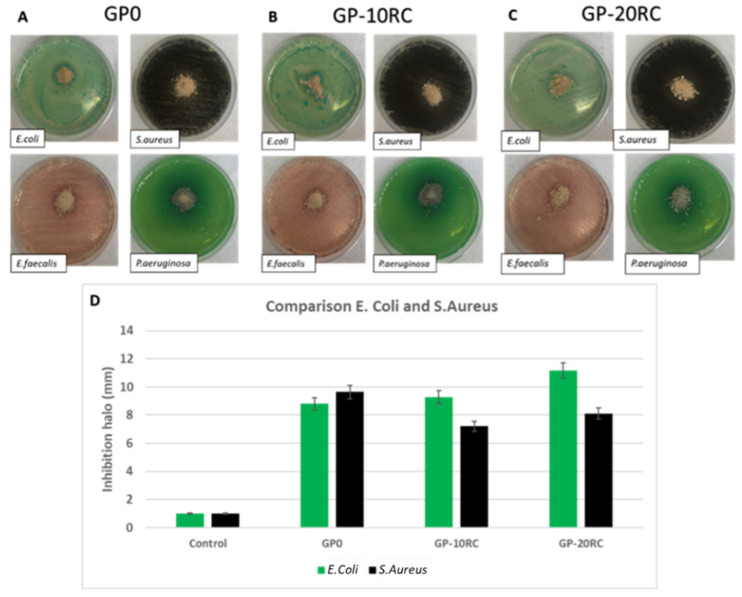
Inhibition halo of *E. coli*, *S. aureus*, *E. faecalis*, and *P. aeruginosa* for samples: (**A**) GP0; (**B**) GP-10RC; and (**C**) GP-20RC. (**D**) Comparison of the inhibition halo of *E. coli* and *S. aureus* for the three geopolymers.

Aiming to estimate the possible antibacterial properties for non-structural building applications (for example glazes on ceramic tiles), both gram-positive and negative bacterial strains are grown in the presence of the synthesized geopolymers. Figure 7A–C reports the images of the plates with the bacteria growth in presence of the geopolymers. As shown by the pictures, there are no evident inhibition halos. This could be explained by the fact that none of the geopolymer samples releases heavy metals in the culture broth at a concentration that negatively affects bacterial growth, which is in accordance with the release test. However, a more detailed observation of the back of Petri plates (images not shown), revealed that *P. aeruginosa* and *E. faecalis* were grown also on the geopolymer samples, while *S. aureus* and *E. coli* were not able to grow on the samples, as reported in Figure 7D. This could be explained by the fact that the formers can resist both possible metals release or pH environment changes [34].

## 4. Discussion

The obtained geopolymeric solids showed good workability in terms of mould filling up capability as well as good chemical stability after 28 d of curing at room temperature. The latter was proved by the absence of efflorescence at the solid surface as well as by the determination of contained increase in the pH and ionic conductivity of the water after immersion with 1:10 = solid:water weight ratio. According to Reference [25], the immersion in water test univocally assess the overall chemical stability of the 3D aluminosilicate network reticulated within the geopolymeric final products when pH are around 9–10 and ionic conductivity is in the range 300–500 mS/m. The presence of unbounded or unreacted –OH^−1^ groups increase the pH upon leaching out from the solid while the Na^+1^ ions increase the water conductivity when the Al^+4^ tetrahedral are not in sufficient number. As underlined by Davidovits [3], presence of the Al^+4^ tetrahedra alternated to Si^+4^ ones with oxygens bridges suffers from unbalanced positive charge that attract the Na^+1^ ions contained in the activator solution. The higher the Al^+4^ tetrahedra number in the 3D geopolymeric network, the higher is the Na^+1^ bonded ions’ number. Reflecting such a structural consideration on the leaching process, the higher the number of leachate Na^+1^ ions, the higher is the ionic conductivity of the distilled water where the solid geopolymer was immersed. The values recorded for the geopolymers of the present studies are in the lower range of the ionic conductivity interval, indicating good stability for all the 3 samples. The sequence that was experimentally recorded for the investigated formulations indicated the higher chemical stability for the sample GP0, followed by GP10RC, and GP20RC, respectively (Figure 2).

Additional mobile species, apart from OH^−1^ and Na^+1^, have been found during the leaching tests (Figure 3). These species are heavy metals, Al^+3^, and Si^+4^. Even though the last two elements are more mobile in the GP0 formulation, their amounts and ionic mobility in solution with respect to Na^+1^ are not sufficient to record an increase in ionic conductivity. Aiming to evaluate the possible life cycle end of the geopolymer and RC samples, the European directives 2006/12/CE and following modifications (2008/9/CE) suggest that the limit value for the recovery of wastes with vanadium is 0.25 ppm, while there are no limits for Al, Ca, and Si. The RC used in this work releases up to 0.01 ppm and according to the obtained data, even though the vanadium release is high in the GP0 sample, the increase of RC content in the geopolymers corresponds to a decrease in vanadium release.

A more direct structural investigation was performed via FT-IR analyses of the solidified samples after 28 d of curing at room temperature (Figure 4). Having chosen a metakaolin-based geopolymer system to which we have added the RC, we focused our investigations on the analysis of the Si-O-T (T = Si or Al) bands at 1080 cm^−1^. The shift of this peak towards lower wavelength is indicative of the efficient reticulation/geopolymerization with the increase of Si-O-Al bonds of the metakaolin amorphous structure [18,26]. The experimental data prove that RC do not seem to affect the geopolymerization process, the principal process reflected in the FT-IR peaks evolution, leaving the Ca-O and Ti-O signal almost untouched. The presence of –OH bonds correspond to the increase in pH of the distilled water when geopolymer samples have been immersed.

The amorphous hump shift in the XRD patterns (Figure 5C) confirm the Si-O-Si to Si-O-Al network of the amorphous fraction of the geopolymeric samples. The presence of the corundum peaks and illite/montmorillonite peaks, clearly visible in the pattern of the sample GP-20RC, indicate the low degree of reactivity of the impurities present in the RC.

Within the 28 d of curing no useful interface between the corundum grains and the geopolymeric matrix was developed to evidence the reinforcement mechanism of the RC inert filler (Figure 6). Being corundum a very hard crystal, we expected the opposite trend in the mechanical performance from GP0 to GP-20RC. The data experimentally collected in the present study proved that the addition of 10 wt% of RC does not affect the GP0 resistance, yet it lowers it instead of increasing it. The lower mechanical performance in presence of RC 10 and 20% could be related to the excess of alkali activator that remains unreacted and prevents proper densification. The absence of a proper densification does not develop a proper matrix/reinforcement interface so that the effect of a hard filler is not exploited.

Combining the intrinsic inertness of the corundum with the absence of heavy metals leaching in water not, presumably, in culture’s broth, the geopolymers added with RC does not show higher antibacterial properties with respect to GP0 (Figure 7). The limited antibacterial properties measured as inhibition halos for the cases of *E. coli* and *S. aureus* show that some bacteria could receive a negative impact on their growth when in contact with the tested materials. This result is not totally negative but could be considered positive when geopolymers are used for applications such as internal protection of sewage pipes.

## 5. Conclusions

The materials formulated in this study showed a number of good results in terms of physical, chemical, and mechanical stability. A sort list can be drafted as follows:

1. fast room temperature consolidation, since after 24 h in plastic mould they were demoulded without deformation;
2. good chemical stability in air, as proved by the absence of efflorescence at the solid surface;
3. good chemical stability in water, as indicated by the contained increase in the pH and ionic conductivity of the water after immersion;
4. the insertion of RC slightly shift the ionic conductivity of water from that of the reference GP0 formulation:ionic conductivity increase from GP0 to GP10RC, and GP20RC;
5. the leaching test results indicated that the release of the heavy metals is below law limits with exception of V, that is present in the starting MK and not in the RC;
6. the principal process of reticulation has been evidenced by the FT-IR peaks analysis that proved that RC do not affect the regular MK’s geopolymerization process;
7. mineralogical analysis did not show any novel crystalline phase formation, remaining the microstructure amorphous for the most fraction of the geopolymeric samples;
8. when introducing RC in the formulations the mechanical performance reaches its maximum compression resistance value with 10% addition, indicating this content as the favoured one; and
9. geopolymers added with RC do not show higher antibacterial properties with respect to GP0 even though some bacteria could receive a slight, yet measurable, negative impact on their growth when in contact with the tested materials.

In conclusion, the results presented above indicate the formation of a stable and safe final product with a ceramic-like appearance.

Future research directions may imply a careful look at the corundum particle/geopolymeric matrix interfaces including reinforcement mechanisms.

In conclusion, the addition of used corundum abrasive powders to geopolymer formulations can de proposed also for wastes deriving from other applications, such as dishing up in free form, drum, vibrating-removal of scrapes, special blasting of different materials and products as well as from grinding and lapping of materials with free grains.

## Figures and Tables

**Table 1:** Chemical composition (XRF) of the as-received corundum abrasive powder.

Al_2_O_3_	SiO_2_	CaO	SO_3_	Fe_2_O_3_	TiO_2_
91.73–92.00%	2.49–3.58%	2.41–2.81%	1.00–1.30%	0.59–1.06%	0.42–0.61%

## Data Availability

The data presented in this study are available on request from the corresponding author.
